# Periodontal health status in cirrhotic patients: a systematic review and meta-analysis

**DOI:** 10.1186/s12903-023-03052-5

**Published:** 2023-06-05

**Authors:** Mengyao Bie, Peiyao Wu, Jieyu Zhou, Yan Li, Lei Zhao

**Affiliations:** 1 Department of Periodontics, West China Hospital of Stomatology, Sichuan University, NO.14 Ren Min South Road 3Rd Section, Chengdu, 610041 Sichuan China; 2 State Key Laboratory of Oral Diseases, National Clinical Research Center for Oral Diseases, Chengdu, China

**Keywords:** Periodontal health status, Liver cirrhosis, Periodontitis, Systematic review, Meta-analysis

## Abstract

**Objectives:**

Liver cirrhosis is a disease with widespread prevalence and high mortality. Oral manifestations, particularly periodontal-related manifestations such as bleeding gums, red and swollen gums, are common in cirrhotic patients but may often be overshadowed by other systemic complications, making them easy to ignore. So this article conducts a systematic review and meta-analysis of periodontal health status in patients with cirrhosis.

**Material and methods:**

We performed electronic searches on the following databases: PubMed, EMBASE, Scopus, Web of Science, and Cochrane Library. Risk of bias evaluation was carried out according to the Fowkes and Fulton guidelines. Meta-analyses were performed with tests for sensitivity and statistical heterogeneity.

**Results:**

Of the 368 potentially eligible articles, 12 studies were included for qualitative analysis, and 9 contributed to the meta-analysis. In terms of periodontal-related parameters, cirrhotic patients presented a greater mean of clinical attachment loss (CAL) (weighted mean differences [WMD] = 1.078, 95% confidence interval [95% CI]: 0.546–1.609, *p* < 0.001), probing depth (PD) (WMD = 0.796, 95% CI: 0.158 to 1.434, *p* = 0.015) and alveolar bone loss (ABL) (WMD = 3.465, 95% CI: 2.946–3.984, *p* < 0.001) than those without, while no statistical difference was found in the papillary bleeding index (PBI) (WMD = 0.166, 95% CI: -0546 to 0.878, *p* = 0.647) and bleeding on probing (BOP) (WMD = 4.913, 95% CI: -3.099 to 12.926, *p* = 0.229). The prevalence of periodontitis was higher in cirrhotic patients than in the control group (odds ratio [OR] = 2.630, 95% CI: 1.531–4.520, *p* < 0.001).

**Conclusions:**

The results indicate that cirrhotic patients have poor periodontal conditions and a higher prevalence of periodontitis. We advocate that they should receive regular oral hygiene and basic periodontal treatment.

**Supplementary Information:**

The online version contains supplementary material available at 10.1186/s12903-023-03052-5.

## Introduction

Liver cirrhosis is a disease with widespread prevalence and high mortality, alcohol misuse, hepatitis virus infection, and nonalcoholic fatty liver disease (NAFLD) are the main causes; the global estimated age-standardized death rate (ASDR) of alcohol-associated cirrhosis was 4.5 per 100,000 [1]. Cirrhosis is a diffuse pathophysiological state of the liver manifested by hepatocyte degeneration and necrosis, hepatic parenchymal fibrosis, regenerative nodules, and finally loss of liver function [2]. Cirrhotic patients often have an imbalance of the coagulation system because of defects in both prothrombotic and antithrombotic components, which can increase gingival bleeding risk [3]. Periodontal pathogenic bacteria, such as *Porphyromonas gingivalis*, *Peurella intermediate*, *Peurella melanobacterium,* and other melanin-producing pathogens are based on hemoglobin, heme as nutrients, bleeding periodontal pocket and provide a better growth environment for related microorganisms [4, 5]. Besides, a reduced immune reaction to bacterial infections owing to immune dysfunction would further drive periodontal infection [6]. Furthermore, defects in Kupffer cells and neutrophil function can turn exaggerated inflammatory responses to systemic inflammation, promoting periodontal disease, impairing periodontal health [7].

Regarding periodontal health status, we must pay attention to the health of periodontal support tissues such as cementum, periodontal ligament and alveolar bone. Clinically, the corresponding indicators are generally used to reflect: probing depth (PD), clinical attachment loss (CAL), alveolar bone loss (ABL), etc. If these periodontal-related clinical indicators are abnormal, the patient may have periodontitis [8]. Periodontitis is a chronic, devastating inflammatory disease caused by bacteria. Severe periodontitis is reported to be the sixth most prevalent medical condition worldwide, placing a huge socio-economic burden on patients [9]. A dysbiosis of the dental plaque biofilm triggers periodontitis, then interacts with the immune defenses of the host, causing destructive inflammatory disorders [10]. Periodontitis not only destroys periodontal health, but also is a significant risk factor for several systemic diseases such as diabetes [11], cardiovascular diseases [12], rheumatoid arthritis [13], and some types of cancers [14]. Periodontitis may regulate the immune and inflammatory response of the body through periodontal pathogens and inflammatory mediators, affecting overall health [15]. Moreover, these systemic diseases may influence the pathogenesis of periodontitis: alter the composition of oral microbiota, increase those species harmful to the balance of microbe-host interaction, and cause a higher inflammatory response, resulting in accelerated periodontal destruction [16, 17].

A recent study showed poor periodontal health in cirrhotic patients, which was significantly associated with three months of hospitalization [18]. Some epidemiological studies revealed that cirrhotic patients presented a significantly higher risk of periodontitis than controls [19, 20]. A recent clinical study also found liver cirrhosis as an independent risk factor for patients with severe periodontitis [21]. The current evidence suggests that liver cirrhosis can likely interfere with the balance of periodontal health, so we conduct a systematic review and meta-analysis about the periodontal health status in cirrhotic patients, providing new evidence for the influence of systemic diseases on periodontal health.

## Materials and methods

### Protocol and registration

The methodology of this systematic review was prepared following the PRISMA (Preference Reporting Requirements for Systematic Review and Meta-Analysis) statement (Table S1). The study has been registered with PROSPERO, number CRD42020199133.

### Eligibility criteria

The criteria were based on the PECOS (participant, exposure, comparison, outcome, study design) method: (1) Participants: the mean age of individuals had to be older than 18 years; (2) Exposure: cirrhotic patients based on histological criteria, clinical signs or laboratory findings to enroll; (3) Comparison: healthy subjects without liver cirrhosis; (4) Outcome: Periodontal clinical parameters related to periodontal health status: Probing depth (PD), clinical attachment loss (CAL), bleeding on probing (BOP), papillary bleeding index (PBI), alveolar bone loss (ABL), or prevalence of periodontitis in the sample population; (5) Study design: included cross-sectional, case–control, and cohort studies; excluded intervention studies, reviews, letters, case reports, and animal studies. No restrictions on language or sample size.

### Search strategy

We conducted an electronic search following the e-bibliographic databases: PubMed, EMBASE, Scopus, Web of Science, and Cochrane Library, searched OpenGray to investigate the gray literature, and analyzed the first 200 hits on a Google Scholar search. The survey included all articles published on or before Jan 18, 2023. Detailed search strategies are provided in Table S2.

To identify any study might be added, a references list of all included articles was manually searched, including *Journal of Clinical Periodontology, Journal of Periodontology, Journal of Periodontal Research, Oral Diseases, Journal of Gastroenterology, Liver International* and *BMC Gastroenterology* up to Jan 18, 2023. The reference manager software (EndNote® X9, Thomson Reuters, New York, USA) was used to group and manage the references.

### Study selection

Two review authors screened titles and abstracts of all identified reports independently to identify potentially eligible studies (PYW and MYB). Obtained full reports when the studies met the inclusion criteria or abstracts provided sufficient data. Two authors independently reviewed the full texts. The final decision about the eligibility of all studies was made by mutual agreement or consultation with the third author (LZ).

### Data extraction

Two reviewers (PYW and MYB) independently extracted information from each study using a standardized data collection form. Recording data were as follows: authors; publication year; origin country; study design; characteristics of the participants (sample size, age, and gender); definition of periodontitis, periodontal measurements; statistical analysis; main findings. The disagreements were handled by reaching a consensus or contacting corresponding authors of the included studies.

### Quality assessment and risk of bias

Used the Fowkes and Fulton checklist [22] to appraise the methodological quality and risk of bias in selected studies. This checklist assessed the quality of the articles following central domains: “Study design appropriate to the objective?”; “Study sample representative?”; “Control group acceptable?”; “Quality of measurements and outcomes?”; “Completeness?”; “Distorting influences?”.

When checking the criteria, we rated each item as a major problem (+ +), a minor problem ( +), no problem (0), or not applicable (NA). The evaluation for each domain put in Table S3. Evaluate the risk of bias through three summary questions: “Bias: Are the results erroneously biased in a certain direction?”, “Confounding: Are there any serious confusing or other distorting influences?” and “Chance: Is it likely that the results occurred by chance?” “YES” and “NO” answers were assigned to each question. If the answer was NO in all questions, the article was at low risk of bias.

### Meta-analysis

Six meta-analyses were performed to evaluate the periodontal health status in cirrhotic patients, including (1) probing depth (PD), (2) clinical attachment loss (CAL), (3) bleeding on probing (BOP), (4) papillary bleeding index (PBI), (5) alveolar bone loss (ABL) and (6) prevalence of periodontitis. The odds ratios (ORs) and 95% confidence intervals (CIs) of studies with dichotomous variables (prevalence) were pooled to estimate the strength of the association between periodontitis and liver cirrhosis. Meanwhile, weighted mean differences (WMDs) together with their 95% CIs for periodontal clinical parameters were calculated to determine their overall effects. Used Cochrane Q chi-squared statistic and I^2^ to examine the potential sources of heterogeneity between the studies, and when detected (Chi-square *p* < 0.05; I^2^ > 50%) the random effects model was preferred [23]. The fixed effects model was chosen to perform the meta-analysis if no statistically significant heterogeneity was observed. If possible, a sensitivity analysis was conducted to explore the extent that inferences might depend on a particular study or number of publications when significant heterogeneity of results was detected across studies. Stata version 14 was used to conduct statistical analyses (Stata Corp, College Station, TX, USA). All reported *p-values* were two-sided at the level of 0.05.

## Results

### Search and selection results

**Fig. 1:**
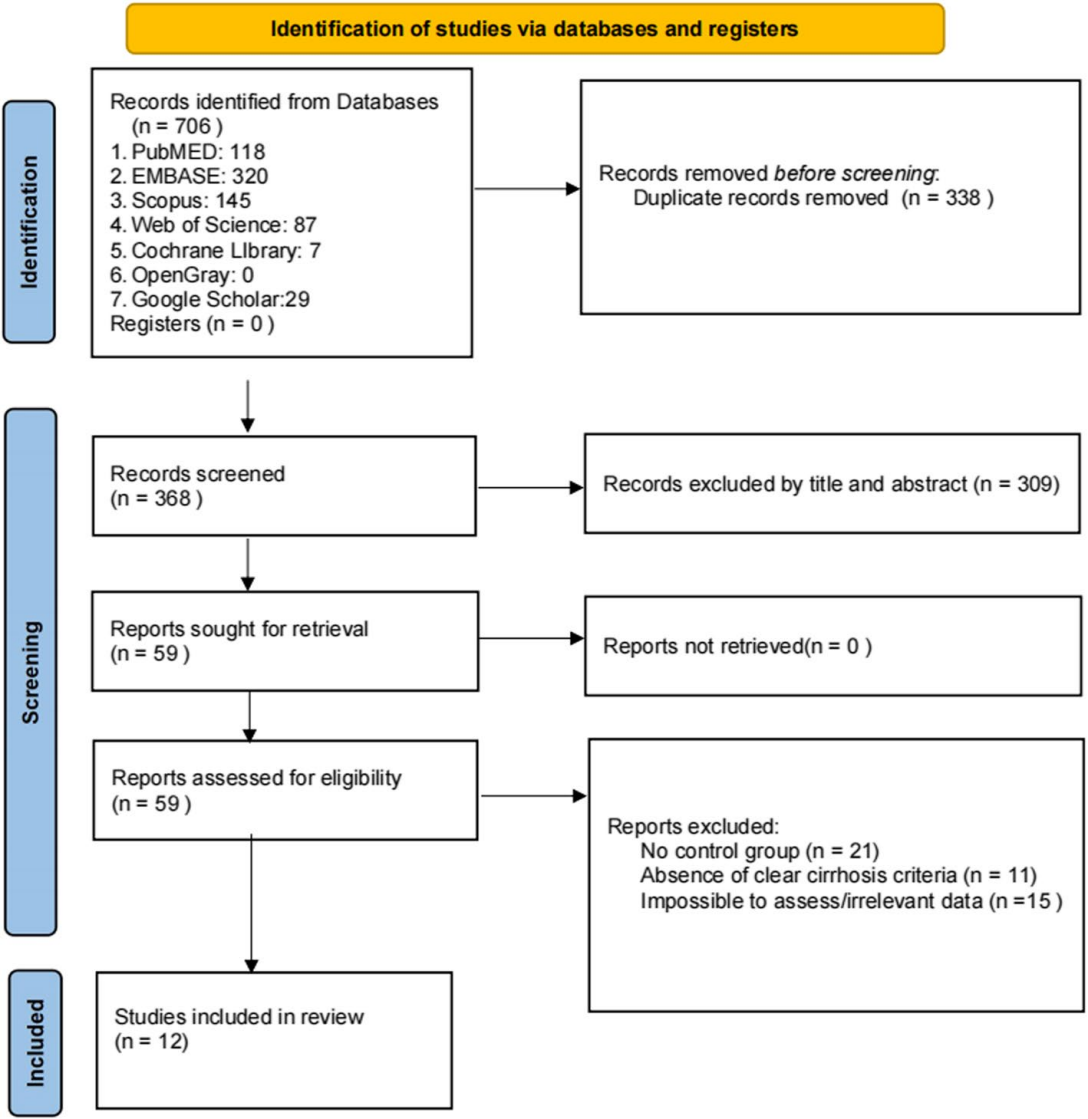
Flow diagram for the study selection procedure according to the PRISMA statement

The search resulted in 118 publications from PubMed, 320 from EMBASE, 145 from Scopus, 87 from Web of Science, 7 from Cochrane Library, 0 from OpenGray, and 29 from Google Scholar. Then, 368 duplicate publications were excluded. 309 publications were removed in the title/abstract screening process. 59 articles were included for full-text appraisal, and 47 were excluded for different reasons (21 did not include comparisons, 11 did not define cirrhosis clearly, 15 did not have relevant data). Finally, 12 articles were included in this systematic review [19, 20, 24–33]. The flow-chart is shown in Fig. 1.

### Characteristics of the Studies

The characteristics of the included studies are shown in Table 1. Among the 12 articles, 4 present case–control design [19, 27, 33, 34], 8 were cross-sectional studies [24–26, 28–32]. The publication year ranged from 1981 to 2021. 3 studies were based in Isarel [25, 29, 30], 2 in Brazil [19, 20], 1 in Iran [24], 1 in Denmark [26], 1 in Austria [28], 1 in Bulgaria [31], 2 in India [27, 32], and 1 in China [33]. The total number of study participants was 1,446, including 604 cirrhotic patients. The age of participants ranged from 18 to 87, and the males varied from 53 to 85%. For the evaluation of periodontal status, the following clinical parameters were applied in most studies [19, 20, 25, 26, 28–32, 35]: PI, GI, PD, CAL, ABL, BOP and PBI. Some also used gingival recession (GR) [20], visible plaque index (VPI) [20], gingival overgrowth (GO) [29], calculus index (CI) [31], Russell’s periodontal index [32], etc. For the prevalence of periodontitis, 3 of the 12 studies [19, 20, 33], involving 842 participants, defined periodontitis: Costa et al. [19] included individuals with moderate, severe and advanced periodontitis according to The 2017 World Workshop Classification system for periodontal and peri-implant diseases and conditions [35]. Di Profio et al. [20] defined periodontitis as 30% or more of teeth with proximal CAL ≥ 5 mm, while Sun et al. [33] defined periodontitis as PD ≥ 5 mm. In these 3 studies, the prevalence of periodontitis in cirrhotic patients ranged from 56% to 85.89%, compared with 18% to 74% in controls.

**Table 1:** The main characteristics of the included studies

Author, year	Country	Study design	Sample size	Mean age	Percentage of males (%)	Definition of periodontitis	Periodontal measurements	Statistical analysis	Main findings
Movin et al. (1981) [26]	Denmark	Cross-sectional	*N* = 73 Cirrhosis: 30 Controls: 43	Cirrhosis: 52.3 ± 1.5 Controls: 51.9 ± 1.1	Overall: 84 Cirrhosis: 83 Controls: 84	NA	Tooth mobility (TM), number of teeth, PI, GI, retentive calculus (RC), CAL	Chi-square test, Mann–Whitney- Wilcoxon test, Student's t-test	Patients suffering from cirrhosis for more than 3 years showed significantly greater loss of attachment, as well as more plaque and calculus compared with those with a disease duration of fewer than 3 years
Novacek et al. (1995) [28]	Austria	Cross-sectional	*N* = 168 Cirrhosis: 97 Controls: 71	Cirrhosis: 31–60 Controls: 21–60	Overall: 65 Cirrhosis: 72 Controls: 52	NA	Number of teeth, PI, CAL	Multiple linear regression, chi-squared test, Kruskall Wallis test (chi-squared approximation), Student's t-test	The presence of cirrhosis is not a predisposing factor for dental and periodontal diseases
Barak et al. (2000) [25]	Israel	Cross-sectional	*N* = 25 Cirrhosis: 8 Controls: 17	Cirrhosis: 49.1 ± 3.90 Controls: 50.16 ± 3.81	NA	NA	ABL	ANOVA	Alveolar bone loss in the advanced liver cirrhosis patients (5.68 ± 0.57 mm) was greater than that in the control group (2.47 ± 0.13 mm)
Oettinger-Barak et al. (2001) [29]	Isarel	Cross-sectional	*N* = 30 Cirrhosis: 13 Controls: 17	Cirrhosis: 46.4 ± 13.34 Controls: 48.53 ± 12.41	Overall: 53 Cirrhosis: 54 Controls: 53	NA	PI, GI, PD, CAL	ANOVA	Liver cirrhosis patients demonstrated greater pocketing and attachment loss compared to healthy controls
Oettinger-Barak et al. (2002) [30]	Isarel	Cross-sectional	*N* = 30 Cirrhosis: 13 Controls: 17	Cirrhosis: 46.4 ± 13.34 Controls: 48.53 ± 12.41	Overall: 53 Cirrhosis: 54 Controls: 53	NA	ABL	ANOVA	Liver cirrhosis patients demonstrated greater bone loss compared to healthy controls
Panov et al. (2011) [31]	Iran	Cross-sectional	*N* = 40 Cirrhosis: 20 Controls: 20	Cirrhosis: 43 ± 2 Controls: 42 ± 2	Overall: 80 Cirrhosis: 85 Controls: 75	NA	PD, CAL, GI, BPI, PI	Mann–Whitney test, t-test	No significant differences between cirrhotic and healthy subjects in terms of periodontal disease parameters
(Banihashemrad et al. (2012) [24]	Bulgaria	Cross-sectional	*N* = 96 Cirrhosis: 25 Controls: 71	Cirrhosis: 24–87 Controls: 18–87	Overall: 63 Cirrhosis: 52 Controls: 66	NA	Number of extracted teeth, debris index (DI), CI, oral hygiene index (OHI), PBI	NA	Patients with chronic hepatitis have poor oral health resulting not only in the large number of extracted teeth, but also and a presence of dental plaque and calculus and gingival bleeding
Raghava et al. (2013) [32]	India	Cross-sectional	*N* = 150 Cirrhosis: 50 Controls: 100	NA	NA	NA	Russell’s periodontal index	ANOVA, Tukey’s HSD test	There is very high statistically significant difference on periodontal destruction in alcoholic liver cirrhosis patients when compared to the control group
Di Profio et al. (2018) [20]	Brazil	Case–control	*N* = 100 Cirrhosis: 50 Controls: 50	Cirrhosis: 52.3 ± 9.6 Control: 51.6 ± 9.3	Overall: 82 Cirrhosis: 82 Controls: 82	30% or more of teeth with proximal CAL ≥ 5 mm	Number of teeth, GR, PD, CAL, BOP, VPI	Paired t-test, McNemar test	Patients with cirrhosis had a greater prevalence of periodontitis than healthy controls (*p* < 0.001). In addition, they had greater mean percentage of sites with CAL ≥ 3 mm (*p* = 0.008) and CAL ≥ 5 mm (*p* = 0.023), greater mean CAL (*p* = 0.003), greater mean gingival recession (*p* < 0.001), and more missing teeth than in the control group (*p* = 0.02)
Costa et al. (2019) [19]	Brazil	Case–control	*N* = 294 Cirrhosis: 98 Controls: 196	Cirrhosis: 49.3 ± 6.9 Controls: 47.12 ± 5.15	Overall: 76 Cirrhosis: 90 Controls: 44	The 2017 World Workshop Classification system for periodontal and peri-implant diseases and conditions [36]	Number of teeth, PD, CAL, PI, BOP	Univariate analysis, multivariate logistic regression	A high prevalence of periodontitis was observed among cases (62.2%) when compared to controls (41.8%). Individuals with cirrhosis presented 2 times greater chances of having periodontitis than controls (OR = 2.28; 95% CI 1.39–3.78; p < .001)
Sun et al. (2021) [33]	China	Cross-sectional	*N* = 320 Cirrhosis: 163 Controls: 140	NA	NA	PD ≥ 5 mm	PD, CAL	ANOVA	The prevalence of periodontitis in cirrhosis patients were 85.3%, which were significantly higher than those in the control group (74.1%, *p* < 0.05). PD and CAL were higher than those in the control group (*p* < 0.01)
Narwat, D et al., (2021) [27]	India	Case–control	*N* = 120 Cirrhosis: 60 Controls: 60	Cirrhosis: 46.63 ± 8.34 Controls: 44 ± 8.04	100%	NA	PI, GI, PD, CAL	ANOVA, post-hoc test	The Plaque Index, gingival index, probing depth and clinical attachment loss has shown a statistically significant difference (*p* = 0.000) when compared between test and control groups. There was no statistically significant difference (*p* = 0.045) found in the number of missing teeth between test and control group

*Abbreviations*: *N* cases with liver cirrhosis or control, *NA* Not Applicable, *CPI* community periodontal index, *GI* gingival inflammation, *OR* odds ratios, *PD* probing depth, *CAL* clinical attachment level, *ABL* Alveolar bone loss, *CAL* clinical attachment level, *PBI* papillary bleeding index, *GI* gingival index, *PI* Periodontal index, ANOVA = analysis of variance, *GR* gingival recession, *VPI* visible plaque index, *AST* aminotransferase, ALT = aminotransferase

### Risk of bias

The risks of bias and quality assessment are included in Table S4. 2 studies were at increased risk of bias because both data had important confounding factors, which possibly caused bias in results [31, 32]. The other 10 presented a low risk of bias while some issues related to assessment criteria (sampling method, sample size, entry criteria/exclusion, definition of controls, source of controls, matching/randomization, comparable characteristics, validity, reproducibility, quality control and confounding factors) reduced the quality of the generated evidence [19, 20, 24–30, 33].

### Meta-analysis for PD

**Fig. 2:**
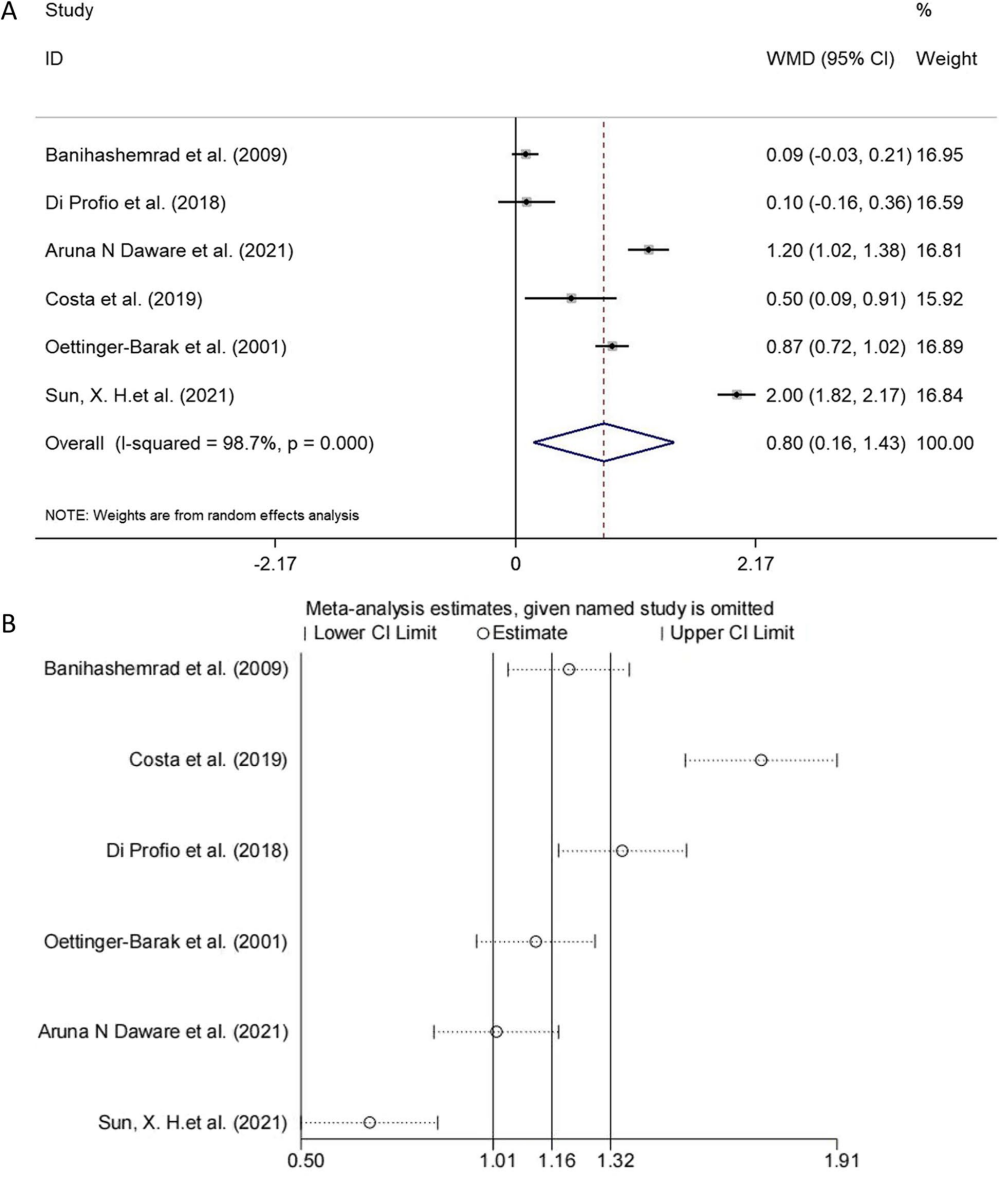
**A** The forest plot of the meta-analysis shows the effect of liver cirrhosis on PD. The data for each study were displayed in the form of weighted mean differences (WMDs) (boxes), 95% CI (horizontal line), and 95% CI for the overall WMD estimate (diamond). **B** Sensitivity analysis demonstrating the influence of each study in the pooled effect of PD. Data are presented as new PD pooled effect amount for each study omission (circles) and 95% CI (horizontal lines)

Six studies were included in the meta-analysis of PD [19, 20, 24, 27, 29, 33]. There was statistically significant heterogeneity among the studies (I^2^ = 98.7%, *p* < 0.001), so the random effects model was used. As shown in Fig. 2A, there were significant differences in the mean PD in cirrhotic patients and control (WMD = 0.796, 95% CI: 0.158 to 1.434, *p* = 0.015). Due to the apparent heterogeneity, we performed a sensitivity analysis to examine the effect of a single study on the overall pooled effects (Fig. 2B), the PD pooled effect amount was 1.16, (95% CI: 1.01 to 1.32), and the removal of Costa et al. from metaregregulation most affected the total pooled effect amount, resulting in a pooled effect amount of 1.71, (95% CI: 1.51 to 1.91).

### Meta-analysis for CAL

**Fig. 3:**
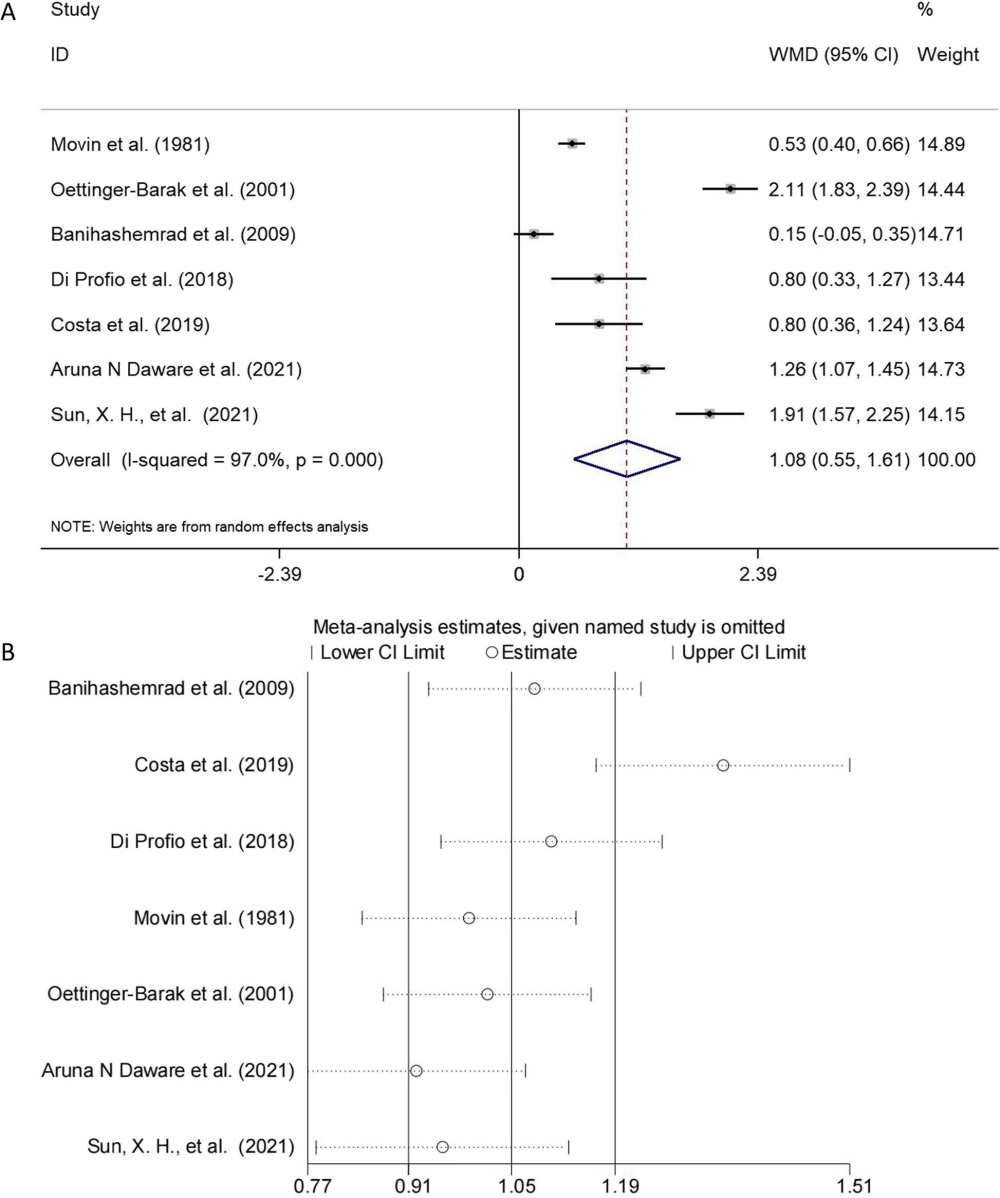
**A** The forest plot of the meta-analysis shows the effect of liver cirrhosis on CAL. The data for each study were displayed in the form of weighted mean differences (WMDs) (boxes), 95% CI (horizontal line), and 95% CI for the overall WMD estimate (diamond). **B** Sensitivity analysis demonstrating the influence of each study in the pooled effect of CAL. Data are presented as new CAL pooled effect amount for each study omission (circles) and 95% CI (horizontal lines)

Eight studies reported CAL of individuals [19, 20, 24, 26–29, 33]. Novacek et al. presented results only in graphics, so the remaining 7 studies were included. The meta-analysis (Fig. 3A) showed that cirrhotic patients presented greater mean of CAL than without (WMD = 1.078, 95% CI: 0.546–1.609, *p* < 0.001). Statistically significant high heterogeneity was observed (I^2^ = 97%, *p* < 0.001). In the sensitivity analysis, coincidentally, only the point estimates of the study in which Costa et al. were removed fell outside the 95% CI of the total effect size (Fig. 3B). When Costa et al. was omitted (WMD = 1.122, 95% CI: 0.531–1.712, *p* < 0.001), the heterogeneity remained high during sensitivity analysis (I^2 =^97.5%).

### Meta-analysis for ABL

**Fig. 4:**
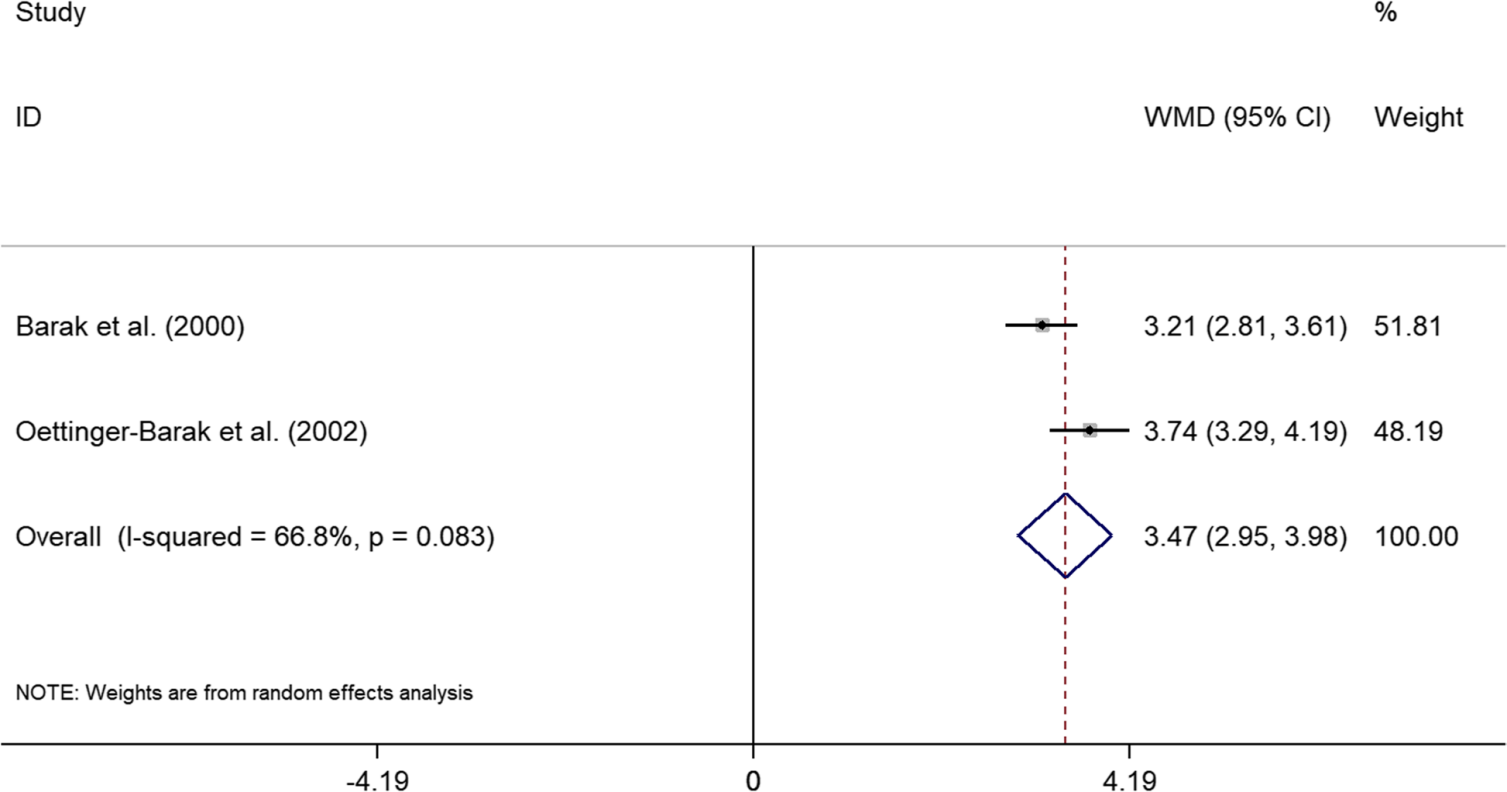
The forest plot of the meta-analysis shows the effect of liver cirrhosis on ABL. The data for each study were displayed in the form of weighted mean differences (WMDs) (boxes), 95% CI (horizontal line), and 95% CI for the overall WMD estimate (diamond)

Two studies reported ABL of individuals [25, 30]. The mean difference in ABL was significant higher in cases compared to controls (Fig. 4) (WMD = 3.465, 95% CI: 2.946–3.984, *p* < 0.001), no statistically significant high heterogeneity was observed (I^2^ = 66.8%, *p* = 0.083). No sensitivity analysis was conducted due to the low number of studies included.

### Meta-analysis for BOP

Two studies reported BOP of individuals [19, 20]. There was no significant difference in mean of BOP (Supplementary Fig. 1) (WMD = 4.913, 95% CI: -3.099 to 12.926, *p* = 0.229), no statistically significant heterogeneity (I^2^ = 46.6%, *p* = 0.171). No sensitivity analysis was conducted due to the low number of studies included.

### Meta-analysis for PBI

Three studies reported PBI of individuals [24, 31, 33]. There was no significant difference in mean of PBI (Supplementary Fig. 2) (WMD = 0.166,95% CI: -0546 to 0.878, *p* = 0.647). And a statistically significant heterogeneity between the studies (I^2^ = 81.1%, *p* = 0.005). No sensitivity analysis was conducted due to the low number of studies included.

### Meta-analysis for prevalence of periodontitis

**Fig. 5:**
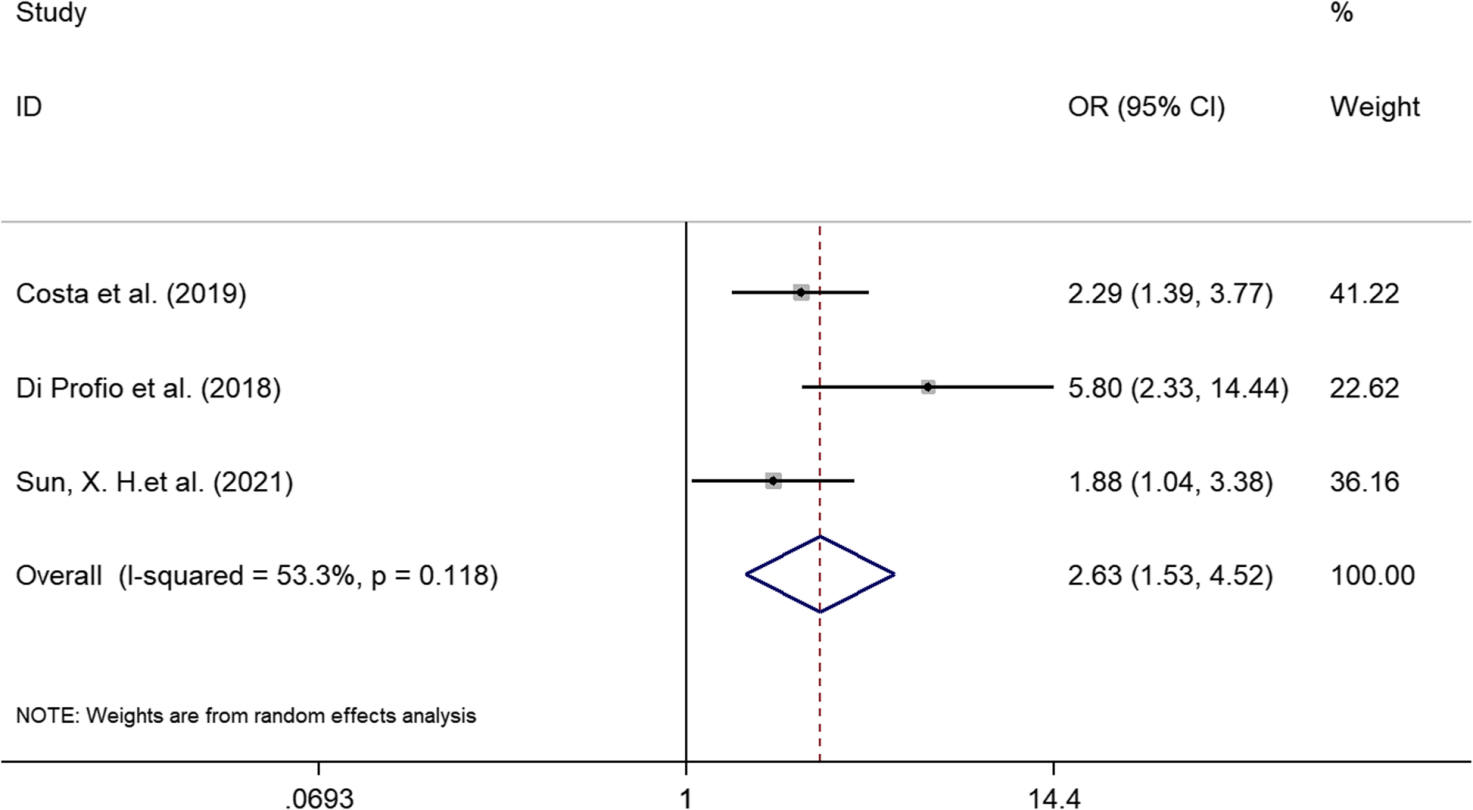
a meta-analysis of the odds ratio of the association between the prevalence of periodontitis and liver cirrhosis was performed. The horizontal line represents the 95% confidence interval (CIs). Diamonds represent the overall odds ratio estimate with its 95% CI

Due to moderate heterogeneity between studies (I^2^ = 53.3%, *p* = 0.118), a random effects model was used. The result of the meta-analysis is depicted in the forest plot in Fig. 5. The pooled ORs of the 3 included studies [19, 20, 33] showed that cirrhotic patients were 2.63-fold more likely than controls to be diagnosed with periodontitis (95% CI: 1.531–4.520, *p* < 0.001).

## Discussion

In clinical diagnosis and treatment, we often find poorer periodontal health in cirrhotic patients, and most of them have symptoms such as bleeding gums, redness, and oral odor. Although the exact mechanism of the link between periodontal inflammation and liver cirrhosis is unclear, some possible hypotheses have been reported: hepatitis virus infections, increased inflammatory response, microbiota dysbiosis, and local microcirculation disorders. First, chronic hepatitis B virus (HBV) and/or hepatitis C virus (HCV) infections are the main causes of liver cirrhosis [36]. Paraschiv et al. found that there was a strong association between chronic HBV, HCV, and periodontitis [37]. HCV-infected patients ranging between 25–44 years old have a poor periodontal condition, manifested increased BOP and PD [38]. Besides, the liver regulates many aspects of the immune system’s physiology and impaired hepatic function can promote a systemic inflammatory state. The levels of serum cytokines were higher in cirrhotic patients, particularly IL-1β, IL-6, IL-8, and TNF-α [39]. These cytokines are also important in the destructive process of periodontal tissues [40]. Therefore, we could speculate that an altered immune response and elevated serum cytokines in cirrhotic patients may precipitate periodontitis. In addition, Jensen et al. analyzed the subgingival microbiome in cirrhotic patients with periodontitis, found that bacteria usually considered as periodontal pathogens: *Porphyromonas gingivalis*, *Tannerella forsythia,* and *Treponema denticola*, three bacteria known as the red complex, showed low abundance. Subgingival microbiota was composed of a unique microbiota not normally associated with periodontitis, suggesting that periodontitis in cirrhotic patients was a consequence of dysbiosis due to a compromised immune system, which might render commensal bacteria to become pathogenic [41]. However, another study reported that cirrhotic patients were at increased risk of developing red complex bacteria [42]. Contrary results make this issue controversial, maybe a high incidence of periodontitis in cirrhotic patients is related to changes in the oral microbial community. Moreover, in recent years a new concept has been proposed–the oral-intestinal-liver axis. This axis triggers hepatic inflammation and exacerbates the systemic inflammatory load through bacterial metabolites, toxins, and multiple inflammatory factors [43]. Furthermore, Funatsu et al. found that the number of capillaries in the periodontium and oral mucosa in cirrhotic patients was lower than those in the controls. This change can cause microcirculatory disturbances, diminish self-defense mechanism of the periodontium and promote periodontal inflammation [44]. Finally, patients with advanced liver disease may have depression [45]. A depressed psychological state of cirrhotic patients can affect oral health care, cause bad oral hygiene, and subsequently precipitate periodontal condition [46]. Depression also leads to dysregulation of neurobiological and neurobehavioral factors, periodontal immune imbalance and microbiome disorders, promoting periodontal inflammation [47].

In this report, we found that the destruction of periodontal support tissue in cirrhotic patients is more serious, the clinical parameters like CAL, PD, and ABL can intuitively reflect these damages, and the prevalence of periodontitis is significantly increased in cirrhotic patients compared to controls. Our conclusions are consistent with a previous systematic review by Grønkjær L et al., they concluded that cirrhotic patients have poor oral hygiene and a high prevalence of periodontitis, periodontitis is related to liver cirrhosis, but since there are not enough articles included, the author only did a qualitative analysis [48]. Therefore, to the authors' knowledge, this article may be the first meta-analysis to quantitatively assess the periodontal status in cirrhotic patients.

Clinical parameters PD, CAL, PBI, BOP, and ABL that are often used to evaluate periodontal health status were chosen to conduct this meta-analysis. PD, ABL, and CAL are the gold standard measures of periodontitis [49]. In this meta-analysis, there was no statistical difference in mean PBI and BOP while the difference in mean PD, CAL, and ABL in cases was significantly higher than in control. In the PD analysis, sensitivity analysis showed that the exclusion of Costa et al. had the greatest impact on the results [19]. We believe the main reason is that the study was relatively new and adopted the 2018 classification of periodontal disease, which only included patients with moderate, severe, and advanced periodontitis [stage II, III, and IV] i.e. The inclusion criteria for PD would be higher than in other studies. PD exhibited high heterogeneity in this meta-analysis, which is undoubtedly related to the inconsistency of the visiting tools used by physicians, whether the visiting tooth position is indexed or full-mouth, the standard of periodontitis, etc. Therefore, future research requires the unification of standards to obtain more valuable data and results. For CAL analysis, the meta-analysis showed that cirrhotic patients presented a greater mean of CAL than without. Statistically significant high heterogeneity was observed, no significant reduction in heterogeneity in sensitivity analysis. However, when the Costa et al. study was ignored, the WMD did not change much, suggesting that the data is valuable for the overall estimate, reflected the periodontal status in cirrhotic patients. The results also showed that cirrhotic patients recorded statistically significant higher mean ABL than controls, no statistically significant high heterogeneity was observed. Gingival bleeding is one of the most common chronic bleeding manifestations in the decompensated stage of liver cirrhosis [50]. Nevertheless, there was no difference in mean BOP, not only because not enough articles, but chronic bleeding from liver cirrhosis affects the measurement of BOP. However, in clinical work we found that when cirrhotic patients with poor periodontal condition receive systematic periodontal basic treatment, the condition of bleeding gums can be improved to a certain extent.

While our results are limited by the small number of studies that meet the criteria for meta-analysis, it is worth noting that cirrhotic patients have poor periodontal health. However, current meta-analysis has limitations, the results should be interpreted with caution: First, the overall quality of the evidence was comparatively low because they were performed in hospitals, which inevitably results in less representation to public. But this limitation is justified, the degree of periodontal inflammation and liver cirrhosis can only be diagnosed in hospitals through specialist examination by physician. Second, studies define and diagnose periodontitis differently, not all studies have chosen consistent techniques to measure periodontitis-related parameters, diagnosing periodontal conditions requires combination of several clinical parameters to be accurate. Using PD, CAL, and ABL to definite periodontitis is widely accepted by periodontists worldwide, according to the recommendations of the European Federation of Periodontology [51]. The absence of recognized criteria to define periodontitis might influence the results, combining using clinical and radiological approaches to define periodontitis are of high quality and safety so that the heterogeneity of studies is reduced and provides more reliable data. Third, the confounding factors, such as age, gender, smoking/drinking history, or systemic status, may have an impact on both cirrhosis and periodontal health. Thus, future well-designed prospective studies to provide strong evidence are necessary. Fourth, our search results were primarily limited to articles published in English. Finally, most studies were cross-sectional or case–control studies, therefore, we cannot draw any causal conclusions from this study. Based on these limitations, we need more longitudinal studies to conduct more comprehensive analyses.

## Conclusion

This article reveals the effect of liver cirrhosis on periodontal health status. Cirrhotic patients tend to have higher CAL, PD, and ABL, the prevalence of periodontitis also increases. However, given the paucity of data and significant limitations, we must interpret the results carefully. Considering our findings, we suggest cirrhotic patients carefully monitor periodontal health and conduct routine periodontal examinations. Dentists and physicians collaborate can improve patients' quality of life and prognosis of liver cirrhosis, and reduce mortality.

## Supplementary Information

**Additional file 1.**

## Data Availability

All data generated and analyzed during this study are included in this published article [and its supplementary information files].

## Abbreviations

CAL: Clinical attachment loss
WMD: Weighted mean difference
95%CI: 95% Confidence interval
PD: Probing depth
ABL: Alveolar bone loss
PBI: Papillary bleeding index
BOP: Bleeding on probing
OR: Odds ratios
NAFLD: Nonalcoholic fatty liver disease
ASDR: Age-standardized death rate
PECOS: Participant, exposure, comparison, outcome, study design
NA: Not applicable
GR: Gingival recession
VPI: Visible plaque index
GO: Gingival overgrowth
CI: Calculus index
PI: Plaque index
GI: Gingival index
