# Supplementary material for: Periodontal health status in cirrhotic patients: a systematic review and meta-analysis
Source: BMC Oral Health. 2023 Jun 5;23:362. doi: 10.1186/s12903-023-03052-5 (PMC10240805; doi:10.1186/s12903-023-03052-5)
Supplement: Supplementary file 1 — Additional file 1. [file 12903_2023_3052_MOESM1_ESM.pdf]

Table S1. PRISMA Checklist.

Section and Topic	Item #	Checklist item	Location where item is reported
**TITLE**			
Title	1	Identify the report as a systematic review.	Page 1
**ABSTRACT**			
Abstract	2	See the PRISMA 2020 for Abstracts checklist.	Page 2
**INTRODUCTION**			
Rationale	3	Describe the rationale for the review in the context of existing knowledge.	Page 1-2
Objectives	4	Provide an explicit statement of the objective(s) or question(s) the review addresses.	Page 2
**METHODS**			
Eligibility criteria	5	Specify the inclusion and exclusion criteria for the review and how studies were grouped for the syntheses.	Page 3
Information sources	6	Specify all databases, registers, websites, organisations, reference lists and other sources searched or consulted to identify studies. Specify the date when each source was last searched or consulted.	Page 3
Search strategy	7	Present the full search strategies for all databases, registers and websites, including any filters and limits used.	Page 3 (Table S2)
Selection process	8	Specify the methods used to decide whether a study met the inclusion criteria of the review, including how many reviewers screened each record and each report retrieved, whether they worked independently, and if applicable, details of automation tools used in the process.	Page 3
Data collection process	9	Specify the methods used to collect data from reports, including how many reviewers collected data from each report, whether they worked independently, any processes for obtaining or confirming data from study investigators, and if applicable, details of automation tools used in the process.	Page 3
Data items	10a	List and define all outcomes for which data were sought. Specify whether all results that were compatible with each outcome domain in each study were sought (e.g. for all measures, time points, analyses), and if not, the methods used to decide which results to collect.	Page 3

Section and Topic	Item #	Checklist item	Location where item is reported
	10b	List and define all other variables for which data were sought (e.g. participant and intervention characteristics, funding sources). Describe any assumptions made about any missing or unclear information.	Page 3
Study risk of bias assessment	11	Specify the methods used to assess risk of bias in the included studies, including details of the tool(s) used, how many reviewers assessed each study and whether they worked independently, and if applicable, details of automation tools used in the process.	Page 4
Effect measures	12	Specify for each outcome the effect measure(s) (e.g. risk ratio, mean difference) used in the synthesis or presentation of results.	Page 4
Synthesis methods	13a	Describe any methods used to synthesize results and provide a rationale for the choice(s). If meta-analysis was performed, describe the model(s), method(s) to identify the presence and extent of statistical heterogeneity, and software package(s) used.	Page 4
Reporting bias assessment	14	Describe any methods used to assess risk of bias due to missing results in a synthesis (arising from reporting biases).	Page 4 (Table S3)
Certainty assessment	15	Describe any methods used to assess certainty (or confidence) in the body of evidence for an outcome.	Page 4
**RESULTS**			
Study selection	16a	Describe the results of the search and selection process, from the number of records identified in the search to the number of studies included in the review, ideally using a flow diagram.	Page 4-5
	16b	Cite studies that might appear to meet the inclusion criteria, but which were excluded, and explain why they were excluded.	Page 5
Study characteristics	17	Cite each included study and present its characteristics.	Page 5 (Table 1)
Risk of bias in studies	18	Present assessments of risk of bias for each included study.	Page 5
Results of syntheses	19a	Present results of all statistical syntheses conducted. If meta-analysis was done, present for each the summary estimate and its precision (e.g. confidence/credible interval) and measures of statistical heterogeneity. If comparing groups, describe the direction of the effect.	Page 5-6
	19b	Present results of all investigations of possible causes of heterogeneity among study results.	Page 5-6

Section and Topic	Item #	Checklist item	Location where item is reported
	19c	Present results of all sensitivity analyses conducted to assess the robustness of the synthesized results.	Page 5-6
**DISCUSSION**			Page 7
Discussion	20a	Provide a general interpretation of the results in the context of other evidence.	Page 7
	20b	Discuss any limitations of the evidence included in the review.	Page 7-8
	20c	Discuss any limitations of the review processes used.	Page 8
	20d	Discuss implications of the results for practice, policy, and future research.	Page 9
**OTHER INFORMATION**			
Registration and protocol	21	Provide registration information for the review, including register name and registration number, or state that the review was not registered.	Page 9
Support	22	Describe sources of financial or non-financial support for the review, and the role of the funders or sponsors in the review.	Page 9
Competing interests	23	Declare any competing interests of review authors.	Page 9

Table S2. Terms used on database search.

Database	Search format
PUBMED	#1 AND #2<br>#1 ((((((((((((((Liver Cirrhosis[MeSH Terms]) OR (Liver Cirrhosis[Title/Abstract])) OR (Cirrhosis[Title/Abstract])) OR (Hepatic Cirrhosis[Title/Abstract])) OR (Cirrhosis, Hepatic[Title/Abstract])) OR (Cirrhosis, Liver[Title/Abstract])) OR (Fibrosis, Liver[Title/Abstract])) OR (Liver Fibrosis[Title/Abstract])) OR (End Stage Liver Disease[MeSH Terms])) OR (End Stage Liver Disease[Title/Abstract])) OR (Chronic Liver Failure[Title/Abstract])) OR (Chronic Liver Failures[Title/Abstract])) OR (Failure, Chronic Liver[Title/Abstract])) OR (Failures, Chronic Liver[Title/Abstract])) OR (Liver Failures, Chronic[Title/Abstract])) OR (Liver Failure, Chronic[Title/Abstract])<br>#2 ((((((((((((((Periodontitis[MeSH Terms]) OR (Periodontitis[Title/Abstract])) OR (Periodontitides[Title/Abstract])) OR (Pericementitis[Title/Abstract])) OR (Pericementitides[Title/Abstract])) OR (Periodontal Diseases[MeSH Terms])) OR (Periodontal Diseases[Title/Abstract])) OR (Disease, Periodontal[Title/Abstract])) OR (Diseases, Periodontal[Title/Abstract])) OR (Periodontal Disease[Title/Abstract])) OR (Parodontosis[Title/Abstract])) OR (Parodontoses[Title/Abstract])) OR (Pyorrhea Alveolaris[Title/Abstract]) OR (Alveolar Bone Loss[MeSH Terms])) OR (Alveolar Bone Loss[Title/Abstract])) OR (Dental Plaque[MeSH Terms])) OR (Dental Plaque[Title/Abstract])
EMBASE	#1 AND #2<br>#1 ('Liver Cirrhosis'/exp) OR ('Cirrhosis':ab,ti) OR ('Hepatic Cirrhosis':ab,ti) OR ('Liver Fibrosis':ab,ti) OR ('End Stage Liver Disease'/exp) OR ('Chronic Liver Failure':ab,ti)<br>#2 ('Periodontitis'/exp) OR ('Periodontal Disease'/exp) OR ('Alveolar Bone Loss'/exp) OR ('Tooth Plaque'/exp) OR ('Periodontal Pocket'/exp) OR ('Periodontal Index'/exp)
SCOPUS	#1 AND #2<br>#1 (TITLE-ABS-KEY(Cirrhosis) OR TITLE-ABS-KEY ("End Stage Liver Disease"))<br>#2 (TITLE-ABS-KEY (Periodontitis) OR TITLE-ABS-KEY ("Periodontal Diseases") OR TITLE-ABS-KEY ("Alveolar Bone Loss") OR TITLE-ABS-KEY ("Dental Plaque"))
WEB OF SCIENCE	#1 AND #2<br>#1 TS=(Cirrhosis) OR TS=(End Stage Liver Disease)<br>#2 TS=(Periodontitis) OR TS=(Periodontal Disease) OR TS=(Alveolar Bone Loss) OR TS=(Dental Plaque)
COCHRANE	#1 AND #2<br>#1 Cirrhosis or " End Stage Liver Disease"<br>#2 Periodontitis or "Periodontal Diseases" or "Alveolar Bone Loss" or "Dental Plaque"
OPEN GREY	Periodontitis and Cirrhosis
GOOGLE SCHOLAR	Periodontitis and Cirrhosis

Table S3. Domains and Risk of Bias are considered in Risk of Bias evaluation according to Fowkes and Fulton.

Guideline	Checklist	Description
Study design appropriate to objectives?	Objective common design<br>Prevalence Cross-sectional<br>Prognosis Cohort<br>Treatment Controlled trial<br>Cause Cohort, case-control, cross-sectional	The type of study was marked in the appropriate type of study. If the type of study was appropriate according to the study design was marked as "0" and as "++" if it was not appropriate.
Study sample representative?	Source of sample	The domain was considered (0) in cases of detailed origin, (+) to specified origin of only one group and (++) in cases of absence of specification of the origin of the groups.
	Sampling method	The item was assigned [0] for a full description of sampling method, [+] for reduced or no explanation of sample method, with no problem in matching between groups, and [++] for poor or no description of sample method, interfering in the matching of the groups.
	Sample size	A minor problem (+) was considered when the sample was not representative or did not report a sample calculation. To a major problem, (++) was considered when no sample calculation was provided and the number of participants was less than 50 participants, (0) was considered in absence of the above factors.
	Entry criteria/exclusion	A minor problem [+] was attributed when the control and case group reported current use of antibiotics or anti-inflammatories, diabetes, smoking or pregnancy. In the case of the presence of more than two previously mentioned items, it was considered as a major problem [++].
	Non-respondents	The (0) was attributed when there was no refusal to participation in the study, (+) was assigned when there was refusal, but did not compromise the sample, and (++) when there was refusal and impairment of the sample size.
Control group acceptable?	Definition of controls	It was attributed (0) when all characteristics of control group were described, (+) when any information was pendent as the origin of control group, the selection criterions and a different origin between case and control groups and (++) when two or more items described in

		previously items.
	Source of controls	It was considered (0) when control group was referred, (+) when the origin of groups was different, but with reasons and (++) when the groups present different origins without reasons.
	Matching/randomization	In this item, (0) was assigned to cases of randomized/matched groups, (+) to cases of no description of randomization, but with matching of groups and (++) to no description of randomization or matching.
	Comparable characteristics	It was attributed (0) to matched groups or not matched by the impossibility of being subsequently adjusted and (++) presence of unpaired variables that were not paired or adjusted.
Quality of measurements and outcomes?	Validity	It was considered (0) when the evaluation method applied is appropriate; (+) when using a single method, but with appropriate sensitivity with good specificity; (++) when using a single method, without an adequate specificity or good sensitivity.
	Reproducibility	It was considered (0) whether the evaluation methods were well described; (+) when a lack description of any step of the method was presented, for example, the identification of the patients of the groups studied in laboratory samples, evaluations at different times or application of different methods between groups of certain pathology; (++) when two or more of the previous items are present.
	Blindness	This item was scored as Not Applicable (NA), due the type of PECO strategy.
	Quality control	It was considered a problem when the examiner was not qualified; a partial periodontal exam was performed [not in all teeth or not in all the six periodontal sites/teeth], the measurement of periodontitis was only radiographic or the absence of the number of evaluated teeth sites. A minor problem [+] was considered when 2 of these characteristics were present, and a major problem [++] if >2 of these characteristics were present.
Completeness	Compliance	It was assigned (0) for a sample size that remains the same from the beginning to the end or decreases without compromising the power of the test; (+) for differences in sample size at the end of the study,

		compromising the power of the test, but with reasons and adjusts; (++) for difference in sample size at the end of the study, compromising the power of the test, without reasons.
	Drop outs	The (0) was scored when there is no loss during the study, (+) when there is withdrawal that involves the inclusion criteria, such as age, sex, (++) when there is withdrawal and it compromises more than one criterion.
	Deaths	This item was scored as Not Applicable (NA), due the type of PECO strategy.
	Missing data	In this item, (0) was assigned to cases of randomized/matched groups, (+) to cases of no description of randomization, but with matching of groups and (++) to no description of randomization or matching.
Distorting influences?	Extraneous treatments	In this item, (0) was considered when there were no external influences; (+) when there are external influences, but that does not interfere in the results; (++) when there are external influences and interferes with the results.
	Contamination	This item was scored as Not Applicable (NA), due the type of PECO strategy.
	Changes over time	In this item, (0) was attributed to data collected in the same time period; (+) to data collected from the control group and the study group at different times that may cause distortions; (++) when the previous item was associated with data from studies already published.
	Confounding factors	Menopausal woman, smokers, diabetics and obese. A minor problem [+] was assigned when 1 or 2 of these characteristics were present and a major problem [++] if there were 3 or more.
	Distortion reduced by analysis	It was considered (0) when it cites the adjustments of the covariates that present distortions; (+) when the article report adjustment, but does not say the criteria; (++) when a distortion was identified, without adjustment.
Summary questions	Bias:<br>Are the results erroneously biased in certain directions?<br>Confounding:<br>Are there any serious confusing or other distorting influences?	“YES” or "NO" answers were assigned for each question. If the answer is NO at the three questions, the article is considered reliable, with a low risk of bias.

Chance:
Is it likely that the results
ocurred by chance?

Table S4. Quality assessment of studies included, according to Fowkes and Fulton.

Guideline	Verification list	Banihashemrad et al. (2009)	Barak et al. (2000)	Costa et al. (2019)	Di Profio et al. (2018)	Movin et al. (1981)	Novacek et al. (1995)	Oettinger-Barak et al. (2001)[30]	Oettinger-Barak et al. (2002)	Panov et al. (2011)	Raghava et al. (2013)	Sun et al. (2021)	Aruna N Daware et al.(2021)
Study design appropriate to objectives?	Objective common design	NA	NA	NA	NA	NA	NA	NA	NA	NA	NA	NA	NA
	Prevalence cross-sectional	NA	NA	NA	NA	NA	NA	NA	NA	NA	NA	NA	NA
	Prognosis cohort	NA	NA	NA	NA	NA	NA	NA	NA	NA	NA	NA	NA
	Treatment controlled trial	NA	NA	NA	NA	NA	NA	NA	NA	NA	NA	NA	NA
	Cause cohort, case-control, cross-sectional	0	0	0	0	0	0	0	0	0	0	0	0
Study sample representative?	Source of sample	0	0	0	0	0	0	0	0	0	++	0	0
	Sampling method	0	0	0	0	0	0	+	+	+	+	0	0
	Sample size	+	+	0	0	0	0	+	+	+	0	0	0
	Entry criteria/exclusion	+	+	+	+	+	++	++	++	++	+	++	+

	Non-respondents	0	0	0	0	0	0	0	0	0	0	0	0
Control group acceptable?	Definition of controls	0	0	0	0	0	+	0	0	0	++	0	0
	Source of controls	0	0	+	+	0	0	0	0	0	0	0	+
	Matching/randomization	0	0	0	0	0	++	0	0	++	++	+	0
	Comparable characteristics	0	0	0	0	0	0	0	0	++	++	++	0
Quality of measurements and omes?	Validity	0	+	0	0	0	0	0	0	0	+	0	0
	Reproducibility	+	0	0	0	0	0	+	+	+	++	0	0
	Blinding	NA	NA	NA	NA	NA	NA	NA	NA	NA	NA	NA	NA
	Quality control	0	+	0	0	0	0	+	+	++	++	+	0
	Compliance	0	0	0	0	0	0	0	0	0	0	0	0
	Drop outs	0	0	0	0	0	0	0	0	0	0	0	0
	Deaths	NA	NA	NA	NA	NA	NA	NA	NA	NA	NA	NA	NA

	Missing data	0	0	0	0	0	0	0	0	0	0	0	0
Distortion influences?	Extraneous treatments	0	0	0	0	0	0	0	0	0	0	0	0
	Contamination	NA	NA	NA	NA	NA	NA	NA	NA	NA	NA	NA	NA
	Changes over time	0	0	0	0	0	0	0	0	0	0	0	0
	Confounding factors	+	+	+	+	+	++	++	++	++	++	+	+
	Distortion reduces by analysis	0	0	0	0	0	0	0	0	0	0	0	0
Summary questions	Bias: Are the results erroneously biased in certain direction?	NO	NO	NO	NO	NO	NO	NO	NO	YES	YES	NO	NO
	Confounding: Are there any serious confusing or other distoring influences?	NO	NO	NO	NO	NO	NO	NO	NO	YES	YES	NO	NO

	Chance: Is it likely that the results ocurred by chance?	NO	NO	NO	NO	NO	NO	NO	NO	NO	NO	NO	NO

NA: Not Applicable

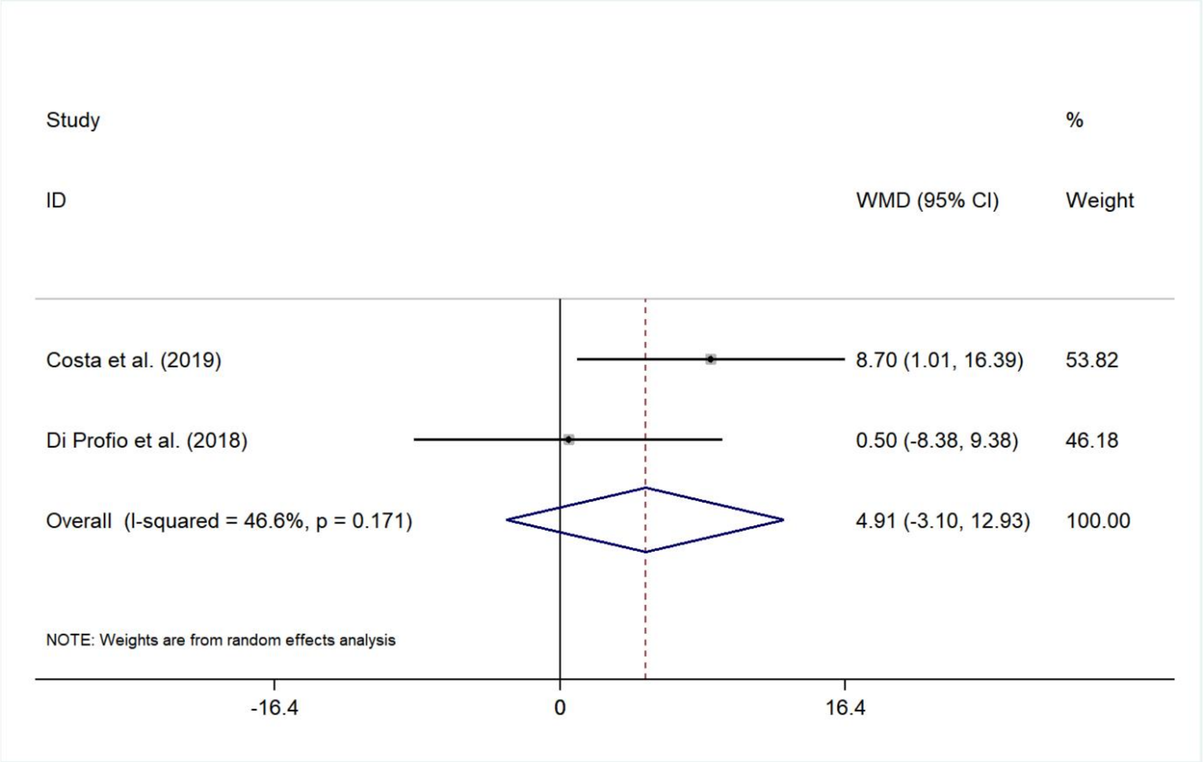

Supplementary Figure 1 The forest plot of the meta-analysis shows the effect of cirrhosis on BOP. The data for each study were displayed in the form of weighted mean differences (WMDs) (boxes), 95% CI (horizontal line), and 95% CI for the overall WMD estimate (diamond).

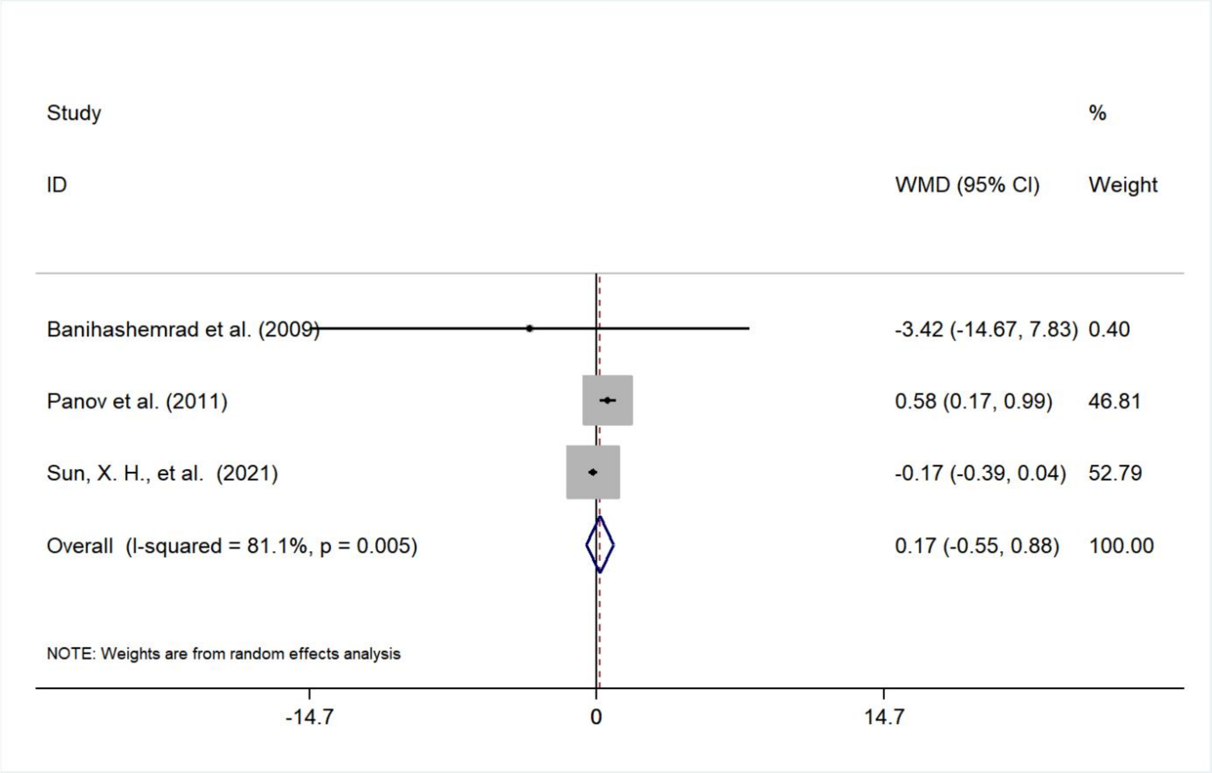

Supplementary Figure 2 The forest plot of the meta-analysis shows the effect of cirrhosis on PBI. The data for each study were displayed in the form of weighted mean differences (WMDs) (boxes), 95% CI (horizontal line), and 95% CI for the overall WMD estimate (diamond).
